# Evidence of Hyperacusis in Adult Rats Following Non-traumatic Sound Exposure

**DOI:** 10.3389/fnsys.2019.00055

**Published:** 2019-10-23

**Authors:** Maryse E. Thomas, Gerson D. Guercio, Kristina M. Drudik, Étienne de Villers-Sidani

**Affiliations:** ^1^Montreal Neurological Institute, McGill University, Montreal, QC, Canada; ^2^Centre for Research on Brain, Language and Music, Montreal, QC, Canada; ^3^Department of Psychiatry, University of Minnesota Medical School, Minneapolis, MN, United States; ^4^Biomedical Sciences Institute, Federal University of Rio de Janeiro, Rio de Janiero, Brazil

**Keywords:** tonotopic map, maladaptive plasticity, tinnitus, hyperacusis, GPIAS, PPI, sound exposure, auditory cortex

## Abstract

Manipulations that enhance neuroplasticity may inadvertently create opportunities for maladaptation. We have previously used passive exposures to non-traumatic white noise to open windows of plasticity in the adult rat auditory cortex and induce frequency-specific functional reorganizations of the tonotopic map. However, similar reorganizations in the central auditory pathway are thought to contribute to the generation of hearing disorders such as tinnitus and hyperacusis. Here, we investigate whether noise-induced reorganizations are accompanied by electrophysiological or behavioral evidence of tinnitus or hyperacusis in adult Long-Evans rats. We used a 2-week passive exposure to moderate-intensity (70 dB SPL) broadband white noise to reopen a critical period for spectral tuning such that a second 1-week exposure to 7 kHz tone pips produced an expansion of the 7 kHz frequency region in the primary auditory cortex (A1). We demonstrate for the first time that this expansion also takes place in the ventral auditory field (VAF). Sound exposure also led to spontaneous and sound-evoked hyperactivity in the anterior auditory field (AAF). Rats were assessed for behavioral evidence of tinnitus or hyperacusis using gap and tone prepulse inhibition of the acoustic startle response. We found that sound exposure did not affect gap-prepulse inhibition. However, sound exposure led to an improvement in prepulse inhibition when the prepulse was a 7 kHz tone, showing that exposed rats had enhanced sensorimotor gating for the exposure frequency. Together, our electrophysiological and behavioral results provide evidence of hyperacusis but not tinnitus in sound-exposed animals. Our findings demonstrate that periods of prolonged noise exposure may open windows of plasticity that can also be understood as windows of vulnerability, potentially increasing the likelihood for maladaptive plasticity to take place.

## Introduction

As recent decades of neuroscience research have revealed the brain’s lifelong capacity for plastic change (Hofer et al., 2006; de Villers-Sidani and Merzenich, 2011), the goal of reopening critical periods (CPs) in order to stimulate learning and recovery in adulthood has become an important area of study. Researchers have already demonstrated the ability to reopen CPs in the auditory (Reed et al., 2011; Zhou et al., 2011; Blundon et al., 2017), visual (Pizzorusso et al., 2002; He et al., 2006; Harauzov et al., 2010), and somatosensory domains (Chung et al., 2017) in animal models. And steps have even been taken in humans, as the histone-deacetylase inhibitor, valproate, was found to reopen a CP for absolute pitch in adult non-musicians (Gervain et al., 2013). The inevitable quest for lifelong adaptability, however, should not be undertaken without considering the potential risks of opening windows of vulnerability on the brain.

One such vulnerability is the opportunity for maladaptive plasticity, which refers to structural or functional nervous system changes that disrupt normal function. Dysplastic symptoms such as hyperexcitation, altered neural connectivity, and topographic reorganizations can interfere with perceptual discrimination (O’Reilly et al., 2019), cause hypersensitivities or phantom percepts (Flor et al., 2001; Costigan et al., 2009; De Ridder et al., 2011), and contribute to chronic pain (Kuner and Flor, 2016). In the central auditory system, maladaptive plasticity is thought to underlie the generation of auditory disorders including chronic tinnitus and hyperacusis, the uncomfortable sensations of ringing in the ears and sound hypersensitivity. These potentially debilitating conditions usually emerge late in life comorbid with hearing loss and affect between 6% to 15% of the general population (Brozoski and Bauer, 2016). Although the exact neural underpinnings of tinnitus and hyperacusis remain elusive, their frequent co-occurrence with hearing loss points to the reduction of auditory inputs as a potential trigger for plasticity in spatially-defined regions of the auditory pathway (Eggermont and Roberts, 2004; Roberts et al., 2010; Langers et al., 2012). In animal models, tinnitus has primarily been associated with expanded representations of mid-to-high frequency regions, hypersynchronization, increased spontaneous firing, and increased burst firing in structures including the cochlear nucleus, inferior colliculus, and auditory cortex (Eggermont and Roberts, 2004; Roberts et al., 2010). Hyperacusis has been related to increased gain in the central auditory pathway in animal models detectable *via* higher spontaneous firing rates and sound-evoked potentials (Sun et al., 2012; Aazh et al., 2014; Hickox and Liberman, 2014). At present, some evidence links spontaneous and sound-evoked hyperactivity to tinnitus or hyperacusis in humans (Adjamian et al., 2009; Gu et al., 2010), but neuroimaging studies have yet to demonstrate macroscopic tonotopic reorganization in patients with tinnitus (Langers et al., 2012; Elgoyhen et al., 2015), illustrating that much remains to be understood in the etiology of both conditions.

Tinnitus has been tentatively linked to lifetime environmental noise exposure (Holgers and Pettersson, 2005; Guest et al., 2017; Moore et al., 2017). In adult rats and cats, prolonged moderate-intensity sound exposures have been shown to produce strong experience-dependent plasticity altering tonotopic organization and auditory excitability (Pienkowski and Eggermont, 2009; Pienkowski et al., 2011; Zhou et al., 2011; Zheng, 2012). We have previously demonstrated that 2 weeks of passive exposure to moderate-intensity white noise can reopen windows of CP-like plasticity in the adult rat auditory cortex (Thomas et al., 2018). We confirmed CP plasticity with a second passive exposure to pure tones that led to the expansion of the corresponding frequency region in the primary auditory cortex (A1). The perceptual consequences of this map expansion are incompletely understood and differ based on the mode of induction, with primarily sound-driven—as opposed to neuromodulatory-driven—expansions impairing discrimination for the exposure frequency (Han et al., 2007; Eggermont, 2013; Froemke et al., 2013). Based on the common phenotype of map expansion in both sound-exposed animals and animals with tinnitus, we wondered if the sound exposure used in our previous study could have imparted our rats with tinnitus or another auditory disorder.

In the present study, we investigated the possibility that cortical map expansion could be indicative of maladaptive plasticity in sound-exposed animals. To this end, we induced 7 kHz map expansion in female adult Long Evans rats using continuous exposure to moderate-intensity (70 dB SPL) broadband white noise for 2 weeks followed by 7 kHz tone pips for 1 week. We hypothesized that this exposure would lead to specific maladaptive plasticity in cortical regions that preferentially respond to 7 kHz accompanied by behavioral evidence of hyperacusis or tinnitus as measured by prepulse inhibition (PPI) and gap-prepulse inhibition (GPIAS) of the acoustic startle reflex, respectively. We documented the effects of exposure on electrophysiological response properties in the A1, anterior auditory field (AAF), and ventral auditory field (VAF). We found evidence of hyperactivity in the AAF of exposed animals consistent with hyperacusis, which was supported by an improvement in PPI when the prepulse was a 7 kHz pure tone. We did not find electrophysiological or behavioral evidence of tinnitus. Our findings indicate that although non-traumatic white noise exposure can open windows of plasticity on the brain, these can also be understood as windows of vulnerability that may increase the likelihood for maladaptive plasticity to occur.

## Materials and Methods

The experimental procedures used in this study were approved by the Montreal Neurological Institute Animal Care Committee and follow the guidelines of the Canadian Council on Animal Care.

### Sound Exposure

Female 3- to 4-month-old Long-Evans rats were housed in sound-attenuated chambers under a 12 h light/dark cycle and given *ad libitum* access to food and water. Rats were assigned to either the naive or sound exposure condition. Naive rats (*N* = 23) had no acoustic manipulation of their environment (background sound level 40 dB SPL). Exposed rats (*N* = 25) were passively exposed to 70 dB SPL (decibels sound pressure level, RMS) continuous white noise for 2 weeks immediately followed by a 1-week exposure to trains of 7 kHz tone pips. From each group, 12 rats were used for behavioral testing (12 Naïve-BEH and 12 Exposed-BEH) while the remaining rats (11 Naive and 13 Exposed) were used for electrophysiological recordings. To reduce the number of animals sacrificed for this study, the electrophysiological data for the Exposed group came from combining two groups of noise + 7 kHz-exposed animals that underwent slightly different 7 kHz exposures. Four rats came from Thomas et al. (2018) and were exposed to 7 kHz pure tones. The other nine rats were exposed to 7 kHz tone pip clouds consisting of pure tones of random frequencies within a $\frac{1}{4}$ octave range centered on 7.6 kHz (ranging between 7 and 8.3 kHz). Other than tone frequencies, all other properties of the tone exposures were the same. The noise and tone pips were generated using custom MATLAB scripts (The MathWorks, Inc., Natick, MA, USA) and played through an Ultralite-mk3 Hybrid Interface (MOTU Inc., Cambridge, MA, USA) with sampling at 192 kHz. The noise stimuli were amplified to a free-field sound level calibrated so that the average stimulus intensity measured in the center of the chamber was 70 dB SPL. Tones were 50 ms in duration (5 ms onset and offset ramps) and delivered in trains of five pips per second. The interval between each train of tones was a random duration generated from a normal distribution with a mean of 2.5 s. The tone pips were amplified to an intensity of 65 dB SPL measured in the center of the chamber. All stimuli were played 24 h per day for the duration of the exposure periods.

### Electrophysiological Recordings

Electrophysiological recordings of the left auditory cortex were performed under isoflurane anesthesia in a shielded soundproof recording chamber. Rats were pre-medicated with dexamethasone (0.2 mg/kg, i.m.) to minimize brain edema. Anesthesia was induced with ketamine/xylazine/acepromazine (63/13/1.5 mg/kg, i.p.) followed by continuous delivery of isoflurane 1% in oxygen *via* endotracheal intubation and mechanical ventilation. Heart rate and blood oxygen saturation were monitored with a pulse oximeter. Body temperature was monitored with a rectal probe and maintained at 37°C with a homeothermic blanket system. Rats were held by the orbits in a custom-designed head holder leaving the ears unobstructed. The cisterna magna was drained of cerebrospinal fluid to further minimize cerebral edema. To access the auditory cortex, the left temporalis muscle was reflected, the skull over the auditory cortex was removed, and the dura was resected. Once exposed, the cortex was maintained under a thin layer of silicone oil to prevent desiccation. Acoustic stimuli were delivered in a free field manner to the right ear through a calibrated speaker. Cortical responses were recorded with a high-impedance 64-channel tungsten microelectrode array (Tucker-Davis Technologies, TDT, Alachua, FL, USA) lowered to a depth of 600–900 μm (layers 4/5). The electrode wires (33 μm diameter) were arranged in an 8 × 8 grid orthogonal to the cortex spaced 375 μm apart with row separation of 500 μm. To maximize recording density, neural responses were consecutively recorded from multiple positions within each rat. The stereotaxic location of each position relative to the first was noted in order to accurately reconstruct auditory maps during offline analysis. Extracellular multi-unit responses were obtained, amplified, and filtered (0.3–5 kHz) using a TDT RZ2 processor. The TDT OpenEx software package was used to generate acoustic stimuli, monitor cortical activity online, and store data for offline analysis.

### Acoustic Stimulation

Frequency-intensity receptive fields were constructed using neuronal responses to a range of frequency-intensity combinations of pure tones. Sixty-six frequencies (0.75–70 kHz; 0.1 octave increments; 25 ms duration; 5 ms ramps) were presented at eight sound intensities (0–70 dB SPL; 10 dB increments) at a rate of one tone per second with three repetitions and in random presentation order. The characteristic frequency (CF) and threshold of a cortical site were defined, respectively, as the frequency and intensity at the tip of the V-shaped tuning curve derived from peri-stimulus time histograms (PSTHs). For flat-peaked tuning curves or tuning curves with multiple peaks, the CF was defined as the frequency with the lowest threshold and the strongest firing rate. Response bandwidths 20 dB above the threshold of tuning curves (BW20) were measured for all sites. The onset latency, defined as the time in ms when the PSTH first exceeded mean baseline firing rate by 2.5 standard deviations, was also measured for each cortical site. The CF, threshold, BW20, and latencies were first determined by an automated custom MATLAB routine and then manually verified by an experimenter blind to the identity of the experimental groups. Cortical sites were identified as belonging to A1, AAF, VAF, or posterior auditory field (PAF) based on published functional characteristics of each field (Polley et al., 2007). These were reversal of tonotopic gradients, onset latencies, threshold, and PSTH morphologies (Supplementary Figure S1). To generate tonotopic maps, Voronoi tessellation was performed using custom MATLAB scripts to create tessellated polygons with electrode penetration sites at their centers.

### Neural Synchrony

The degree of neural synchronization in the auditory cortex was computed from recordings of spontaneous neural activity that were at least 5 min long. Recordings with apparent burst suppression were not included in analyses. Burst suppression was characterized by periods of high spontaneous firing alternating with periods of no activity determined through visual inspection of the raster plots and continuous average firing rate. If a portion of any recording was deemed to have burst suppression, the recording was rejected. The average coefficient of variation (CV) of the inter-spike interval—a measure of burstiness—corresponded well with our classification of burst suppression, as the mean CV was significantly higher for recordings identified as having burst suppression (mean = 3.45, SD = 0.15) than those that were not [mean = 1.86, SD = 0.07; mixed-effects one-way analysis of variance (ANOVA) *F*_(1,48.13)_ = 93.34, *p* < 0.0001, *n* = 2,070 units within 53 positions and 24 rats]. Offline spike sorting was performed using TDT OpenSorter software to isolate single unit activity based on an automated Bayesian sorting algorithm. The success of the spike sorting algorithm was assessed by inspecting the number of refractory period violations for all identified clusters (Supplementary Figure S2). The fraction of spikes that fell within a 2 ms refractory period was calculated and it was found that 36.1% of all clusters had zero refractory period violations and 96.9% of all clusters had two or fewer violations per 100 spikes (Supplementary Figure S2A). An average of 1.63 units was identified per electrode channel. Example histograms of the interspike interval and the autocorrelation of spike times for representative units are presented in Supplementary Figure S2B, displaying a dearth of spikes occurring within the refractory period. In addition, the percentage of refractory period violations did not differ between experimental groups (Supplementary Figure S2C). These results indicate that there are a relatively small number of false-positive classifications present in the data, which are unlikely to affect experimental outcomes. Measures of synchronization were computed from binary spike events detected from A1 units in separate channels. Cross-correlograms were computed by counting the number of spike coincidences for pairs of spike trains for time lags of −500 to 500 ms with 1 ms bin size and normalized by dividing each bin by the square root of the product of the number of total discharges in each spike train (Eggermont, 1992).

### Behavioral Testing

Behavioral testing took place during the day at the Glen Site of the McGill University Health Centre. At the end of the exposure period, each Naïve-BEH animal was randomly assigned to a pair with one Exposed-BEH animal. Once paired, the rats were transported in their original cages to a loading area by cart. There, they were transferred to a vehicle and driven to the Glen Site, approximately 25 min away. The rats were again transported to a holding area adjoining the behavioral testing facility by cart where they were acclimatized for a minimum of 2 h. Rats remained covered for all of the steps above until they reached the holding area. The paired rats then underwent behavioral testing simultaneously in order of pairing (two rats were tested at a time). This procedure took place twice, with six animals from each group tested on each day. All behavioral data were collected in sound-attenuating chambers. Sounds were delivered from a free-field speaker and rats were free to roam the chamber. The acoustic startle response was measured using the LE 118–8 Startle and Fear Interface (Panlab, Barcelona, Spain). The startle pulse was a white noise burst (120 dB SPL, 40 ms) for both GPIAS and PPI. For GPIAS, rats were acclimatized for 3 min in a pure tone background that was either 3.5 kHz or 7 kHz (65 dB SPL), followed by four randomly interleaved no-gap and gap (30 ms) trials (intertrial interval 12–30 s). During the gap trials, the gap preceded the pulse by 60 ms. This procedure was performed three times, and startle activity for the no-gap and gap trials were averaged across a total of 12 trials each. For PPI, rats were acclimatized for 3 min in a white noise background (65 dB SPL). The subsequent experimental protocol consisted of 10 trials each of no stimulus, the startle pulse alone, and two prepulse frequencies (3.5 or 7 kHz, 20 ms, 75 dB SPL) presented 60 ms before the startle pulse, in pseudorandom order (intertrial interval 12–30 s). The startle activity for the no stimulus, startle pulse, 3.5 kHz prepulse, and 7 kHz prepulse trials were averaged across the 10 trials. We calculated prepulse inhibition of the startle response using the formula: %PPI = 100 − (startle response for prepulse trials/startle response for startle pulse alone trials) × 100. We calculated gap-prepulse inhibition of the startle response with the formula: %GPIAS = 100 − (startle response for gap trials/startle response for no-gap trials) × 100.

### Statistical Analyses

For all statistical analyses, results are reported in parentheses including test name, statistic, and number of data points per level of nested data. Linear mixed-effects models (Reed and Kaas, 2010; Aarts et al., 2014) were used to analyze data collected through nested experimental designs (e.g., for synchronization analyses: neuron pair nested within recording position nested within rat). For these models, recording position nested within rat ID were included as random effects. A matched pairs design using paired *t*-tests was employed to analyze behavioral data in order to control for potential confounding effects of transport, handling, waiting, and testing times on the acoustic startle response (Geyer and Swerdlow, 1998; Longenecker and Galazyuk, 2012). Accordingly, the effect size calculated by Cohen’s dav is reported for behavioral results (Lakens, 2013). Analyses were conducted using MATLAB and JMP 13 (SAS Institute, Cary, NC, USA). The mixed-effect test results are reported with the degrees of freedom denominator approximated for normal data using the Kenward-Roger adjustment. Unless otherwise stated, Tukey’s test evaluated at an alpha level of 0.05 was used for all *post hoc* comparisons. Where applicable, back-transformed means derived from statistical models were plotted in figures. Where results are not shown in figures, means ± standard error are reported in the text.

## Results

### Electrophysiological Correlates of Sound Exposure

**Figure 1:**
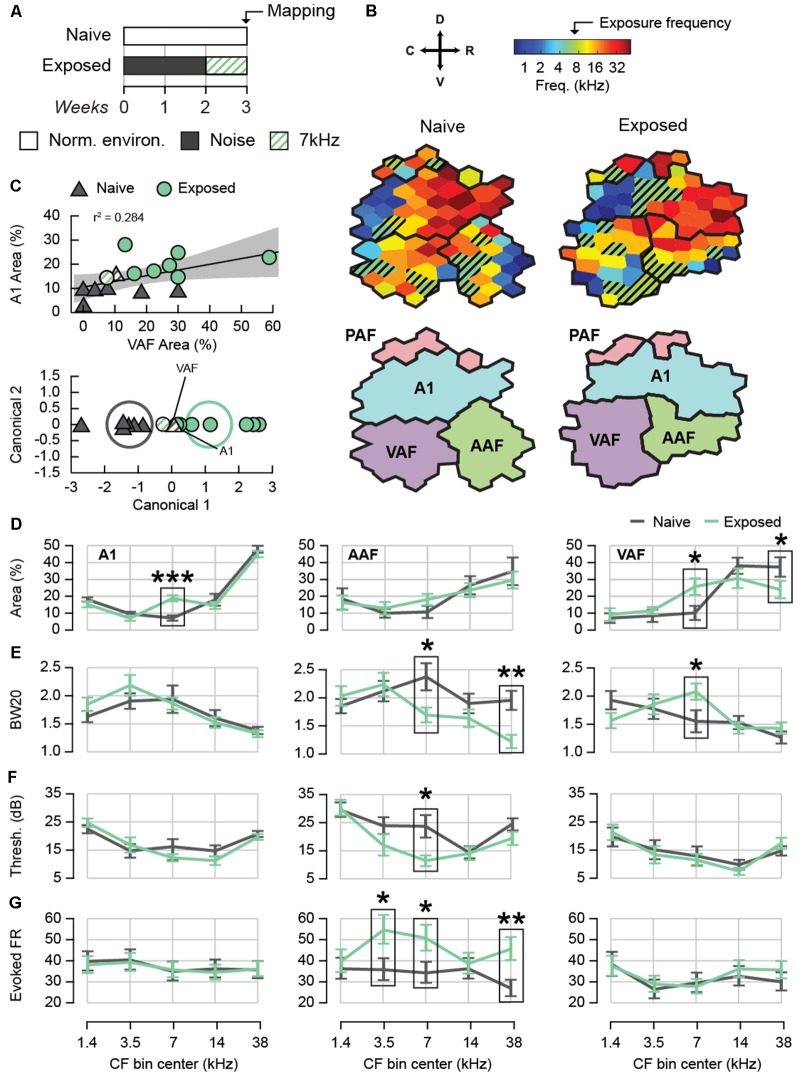
Effect of sound exposure on cortical tuning and tone-evoked activity. **(A)** Sound exposure protocol. Naïve rats were housed in a normal acoustic environment while exposed rats were passively exposed to 2 weeks of moderate-intensity broadband white noise followed by 1 week of 7 kHz tone pips. **(B)** An example characteristic frequency (CF) map from each experimental group containing all auditory fields. Hatched sites represent those with a CF of 7 kHz ± $\frac{1}{2}$ octave. **(C)** Top: correlation between the percent A1 area and percent VAF area with a CF of 7 kHz ± $\frac{1}{2}$ octave. Shaded region represents 95% confidence of fit for the regression. Bottom: canonical plot of the linear discriminant analysis based on the percent of A1 and VAF area with a CF of 7 kHz ± $\frac{1}{2}$ octave. Rats were automatically classified as either naïve or exposed; hatched points identify rats that were misclassified. Ellipses represent the 95% confidence region for the true mean of each group. **(D)** Average map area with CF in five frequency bins. Only full auditory fields were used for map percentages. **(E)** Average BW20 for receptive fields with CF in five frequency bins. **(F)** Average cortical threshold for receptive fields with CF in five frequency bins. **(G)** Average tone-evoked firing rate for units with CF in five frequency bins. *p < 0.05, **p < 0.01, ***p < 0.001. Error bars represent standard error of the mean (SEM). A1, primary auditory field; AAF, anterior auditory field; VAF, ventral auditory field; PAF, posterior auditory field. See Table 1 for number of rats, recording positions, and cortical sites per auditory field and group.

We documented the effects of 2 weeks of passive exposure to white noise followed by 1 week of 7 kHz tone pip exposure on electrophysiological response properties in 13 rats (Exposed Group) and compared them to 11 rats that were housed in a standard acoustic environment (Naïve Group; Figure 1A). CF tonotopic maps were reconstructed from the left auditory cortex under isoflurane anesthesia using *in vivo* multiunit responses to presentations of tone pips of various frequencies and intensities (Figure 1B). Responsive sites were classified as belonging to A1, AAF, VAF, or PAF based on the published functional characteristics of each field (Polley et al., 2007; Profant et al., 2013), specifically reversal of tonotopic gradients, onset latencies, threshold, and PSTH morphologies (Supplementary Figure S1). Using functional properties alone, we were not able to distinguish VAF from the fifth rat auditory field, suprarhinal auditory field (SRAF), so any presumed VAF or SRAF site was classified under the common label of VAF. In addition, we did not conduct analyses on the data we obtained from PAF due to the difficulty of assigning a CF to most PAF units, which have broad and noisy tuning curves. For each animal, we determined whether we obtained full or partial A1, AAF, and VAF maps. A full map was defined by having low, medium, and high frequency regions as well as a reversal of the tonotopic gradient on one border and non-auditory sites on the opposite border. In Figure 1B, the representative CF maps from each group were selected for having full maps of each field. Table 1 lists the number of cortical sites obtained for each auditory field and experimental group for both full and partial maps.

**Table 1:** Summary of data.

					Sites/Units per CF bin					
	Group	Field	Rats	Positions	1.4	3.5	7	14	38	Total
**All data**	Naive	A1	10	22	55	29	21	52	155	312
		AAF	11	22	31	16	19	35	50	151
		VAF	10	18	15	15	17	48	52	147
		PAF	10	17	-	-	-	-	-	69
		***All***	***11***	***24***						***679***
	Exposed	A1	13	30	47	29	67	59	187	389
		AAF	11	23	23	17	27	31	43	141
		VAF	13	27	19	21	46	62	47	195
		PAF	11	21	-	-	-	-	-	105
		***All***	***13***	***30***						***830***
**Full fields only**	Naïve	A1	10	21	53	28	21	52	136	290
		AAF	7	16	22	15	18	32	44	131
		VAF	7	12	11	13	12	43	42	121
		PAF	5	10	-	-	-	-	-	49
		***All***	***11***	***24***						***591***
	Exposed	A1	9	19	47	21	59	47	141	315
		AAF	8	19	23	17	25	27	37	129
		VAF	9	19	17	20	41	49	39	166
		PAF	8	18	-	-	-	-	-	99
		***All***	***13***	***30***						***709***
**Sorted units**	Naïve	A1	10	19	77	32	34	77	197	417
		AAF	11	19	33	23	18	29	65	168
		VAF	9	15	17	17	17	62	76	189
		PAF	10	17	-	-	-	-	-	110
		***All***	***11***	***20***						***884***
	Exposed	A1	13	25	76	36	90	80	263	545
		AAF	11	18	28	17	28	36	45	154
		VAF	13	22	25	33	62	91	59	270
		PAF	10	18	-	-	-	-	-	164
		***All***	***13***	***25***						***1,133***

*Number of rats and recording positions from which data were obtained for each auditory field and experimental group. Number of cortical sites (or units, for sorted data) per CF bin in each field. PAF units were not assigned CFs*.

We first compared the degree of 7 kHz map expansion between exposed and naïve animals (Figures 1B–D). Using full field maps only, we calculated the percentage of map area with CFs in five frequency bins with centers at approximately 1.4, 3.5, 7, 14, and 38 kHz. The range of each bin was 1 octave except for the first and last bin, which were 1.7 and 1.8 octaves respectively. The bins were defined in relation to 7 kHz in order to maximize specificity for the middle bins while covering the full range of recorded CFs. In A1, as expected, we observed a significantly greater percentage of map area tuned to 7 kHz for the Exposed group (two-way ANOVA with Group and Bin as factors. Interaction *F*_(4,4)_ = 5.30, *p* = 0.0007 followed by simple main effects for 7 kHz *F*_(1,85)_ = 16.94, *p* < 0.0001, *n* = 19 rats). This over-representation was not compensated by a consistent under-representation in another frequency bin as no other simple main effect was significant (*F*_(1,85)_ ≤ 1.41, all *p* ≥ 0.2387). In AAF, we detected no difference in map area for any frequency bin (two-way ANOVA with Group and Bin as factors. Interaction: *F*_(4,4)_ = 0.55, *p* = 0.6965, *n* = 15 rats), whereas in VAF we observed a significant over-representation of the 7 kHz frequency bin for exposed animals, as well as a significant decrease in map area in the highest frequency bin (two-way ANOVA with Group and Bin as factors. Interaction *F*_(4,4)_ = 2.96, *p* = 0.0257 followed by simple main effects for 7 kHz *F*_(1,70)_ = 5.85, *p* = 0.0182 and 38 kHz *F*_(1,70)_ = 4.30, *p* = 0.0418, *n* = 16 rats). No other frequency bin was significantly changed (simple main effects *F*_(1,70)_ ≤ 1.33, all *p* ≥ 0.2521). To ensure that we did not oversample the 7 kHz frequency region in the Exposed group, we compared the average distance between each site and its nearest neighbor from full field maps. We observed no significant differences in nearest-neighbor distance between Naïve and Exposed animals in any frequency bin for any field, confirming that differences in frequency representation were not due to differences in sampling (mixed-effects two-way ANOVAs with Group and Bin as factors. A1: mean distance Naïve = 323.72 ± 6.82 μm, Exposed = 326.21 ± 7.14 μm, Interaction *F*_(4,579)_ = 1.00, *p* = 4060. Main effect of Group *F*_(1,17.26)_ = 0.06, *p* = 0.8038, *n* = 605 sites within 19 rats. AAF: mean distance Naïve = 336.20 ± 11.56 μm, Exposed = 329.73 ± 10.77 μm, Interaction *F*_(4,246.8)_ = 1.04, *p* = 0.3863. Main effect of Group *F*_(1,13.31)_ = 0.17, *p* = 0.6887, *n* = 260 sites within 15 rats. VAF: mean distance Naïve = 355.58 ± 11.85 μm, Exposed = 338.55 ± 10.09 μm, Interaction *F*_(4,269.5)_ = 1.73, *p* = 0.1445. Main effect of Group *F*_(1,14.9)_ = 1.20, *p* = 0.2911, *n* = 287 sites within 16 rats). The above results document for the first time that noise-induced CP plasticity extends to A1 and VAF, but not AAF.

It is possible that not every sound-exposed rat will exhibit CP-like plasticity. However, if 7 kHz map expansion is a reliable indicator, it could be used to distinguish rats that show phenotypic CP plasticity from those that do not. We explored this possibility using a linear discriminant analysis to test the hypothesis that exposed and naive rats could be distinguished based on a linear combination of the 7 kHz percent map areas in more than one auditory field (Figure 1C). Only animals with full maps in both A1 and VAF were included (7 Naïve and 8 Exposed). The 7 kHz percent map area in A1 and VAF were positively correlated, *r* = 0.53, *p* = 0.0408, *n* = 15 rats (Figure 1C, top). This is in contrast to A1 and AAF, *r* = 0.09, *p* = 0.8068, *n* = 10 rats, and VAF and AAF, *r* = 0.22, *p* = 0.5964, *n* = 8 rats, which were not significantly correlated. The canonical function resulting from the linear discriminant analysis was statistically significant (canonical correlation = 0.79, Wilks’ Lambda = 0.38, *F*_(2,12)_ = 9.68, *p* = 0.0031, *n* = 15 rats, Figure 1C, bottom). Reclassification of the rats based on the new canonical variable using leave-one-out cross-validation was successful: 88.10 ± 1.2% of the rats were correctly classified into their exposure condition. The canonical function was positively correlated with both 7 kHz percent map area in A1, *r* = 0.99, *p* < 0.0001, *n* = 15 rats, and VAF, *r* = 0.66, *p* = 0.0069, *n* = 15 rats. This result was approximately equivalent to performing a linear discriminant analysis using the 7 kHz percent map area in A1 alone and better than using VAF alone. When including only A1, the canonical function was significant (canonical correlation = 0.77, Wilks’ Lambda = 0.41, *F*_(1,17)_ = 24.15, *p* = 0.0001, *n* = 19 rats), cross-validated reclassification led to 89.47 ± 0.41% correct classification. When including only VAF, the canonical function was also significant but less successful (canonical correlation = 0.53, Wilks’ Lambda = 0.72, *F*_(1,14)_ = 5.38, *p* = 0.0360, *n* = 16 rats). Cross-validated reclassification led to 71.25 ± 1.4% correct classification. These results show that the degree of map expansion is relatively consistent within each animal; rats with high map expansion in A1 are likely to have high map expansion in VAF. This characteristic also allows rats that have undergone sound exposure to be classified with high accuracy, suggesting that degree of map expansion is a reliable indicator of CP plasticity whether taking into account only A1 or A1 and VAF together.

To establish the electrophysiological correlates of 7 kHz map expansion, we continued to compare neural response properties in five CF bins using data from both full and partial maps. We predicted that the 7 kHz-tuned neurons of exposed animals would show additional evidence of plasticity. We compared the receptive field bandwidth 20 dB above threshold (BW20), a measure of response specificity, in each auditory field (Figure 1E). In A1, we observed no significant change in BW20 following exposure for any CF bin (mixed-effects two-way ANOVA with Group and Bin as factors. Interaction *F*_(4,687.7)_ = 1.10, *p* = 0.3545. Main effect of Group *F*_(1,70.18)_ = 0.26, *p* = 0.6093, *n* = 701 sites within 52 positions and 23 rats). In AAF we found significantly narrower BW20s for the 7 kHz and 38 kHz bins (mixed-effects two-way ANOVA with Group and Bin as factors. Interaction *F*_(4,278.1)_ = 3.32, *p* = 0.0113 followed by simple main effects for 7 kHz *F*_(1,194.5)_ = 6.25, *p* = 0.0132 and 38 kHz *F*_(1,127.9)_ = 11.94, *p* = 0.0007. No other CF bin was significant *F*_(1,160.3–234.5)_ ≤ 1.42, all *p* ≥ 0.2360, *n* = 292 sites within 47 positions and 22 rats). In VAF, on the other hand, we observed broader BW20s for the 7 kHz bin (mixed-effects two-way ANOVA with Group and Bin as factors. Interaction *F*_(4,327.1)_ = 2.74, *p* = 0.0288, followed by simple main effects for 7 kHz *F*_(1,242.8)_ = 5.42, *p* = 0.0207. No other CF bin was significant *F*_(1,121.5–278.7)_ ≤ 2.78, all *p* ≥ 0.0966, *n* = 342 sites within 45 positions and 23 rats). These differences demonstrate a reduction in tuning specificity for VAF neurons tuned to 7 kHz following sound exposure and an increase in specificity for AAF neurons tuned to 7 kHz and 38 kHz.

Cortical thresholds measure a neuron’s sensitivity to low intensity sounds and can provide an approximate estimate of hearing thresholds. We compared the average cortical thresholds of neurons in each CF bin between experimental groups (Figure 1F). We observed no group differences in A1 or VAF for any CF bin (mixed-effects two-way ANOVAs with Group and Bin as factors A1: Interaction *F*_(4,657)_ = 1.28, *p* = 0.2761. Main effect of Group *F*_(1,49.79)_ = 0.01, *p* = 0.9190, *n* = 701 sites within 52 positions and 23 rats. VAF: interaction *F*_(4,310.9)_ = 0.71, *p* = 0.5867. Main effect of Group *F*_(1,43.54)_ = 0.02, *p* = 0.8924, *n* = 342 sites within 45 positions and 23 rats). In AAF, however, we found that average thresholds were significantly lower for the 7 kHz bin (mixed-effects two-way ANOVA with Group and Bin as factors. Interaction *F*_(4,255.6)_ = 2.41, *p* = 0.0494 followed by simple main effects for 7 kHz (*F*_(1,124.8)_ = 4.70, *p* = 0.0320. No other CF bin was significant *F*_(1,67.93–156.3)_ ≤ 2.28, *p* ≥ 0.1334, *n* = 292 sites within 47 positions and 22 rats). These results show that after sound exposure, AAF became more sensitive to the 7 kHz frequency. Importantly, the cortical thresholds of the Exposed group were either the same or lower than Naïve for all fields, demonstrating that the exposure intensities were non-traumatic and did not cause any apparent hearing loss. Taken together, the changes in BW20 and cortical thresholds observed in sound-exposed animals may highlight differences in the receptive field properties of AAF and VAF. VAF neurons tend to have narrow tuning curves with low thresholds while AAF neurons tend to have broad tuning curves with relatively high thresholds. Plasticity following sound exposure appears to have reduced these field-specific qualities for 7 kHz-tuned neurons.

Sound-evoked firing rates are elevated in hyperacusis (Sun et al., 2012; Aazh et al., 2014; Hickox and Liberman, 2014). We compared the tone-evoked firing rate between exposed and naive animals (Figure 1G). The average firing rate in response to the full range of tonal stimuli (66 frequencies presented at eight intensities) was considered. The rate was computed from the number of spikes counted between 8 and 58 ms after tone presentation minus the number of spikes counted in the 50 ms preceding tone presentation. As could be expected, firing rate was positively correlated with sound intensity, *r* = 0.11, *p* < 0.0001, *n* = 7,093 observations. We also found that onset latency was negatively correlated with firing rate, *r* = −0.20, *p* < 0.0001, *n* = 7,093 observations, possibly because a fixed epoch window resulted in less spikes being counted for sites with later latencies. We did not observe a significant difference in onset latency between naïve and exposed animals for any field (mixed effects two-way ANOVAs with Group and Bin as factors. A1: Interaction *F*_(4,678.3)_ = 1.74, *p* = 0.1403. Main effect of Group *F*_(1,64.63)_ = 0.39, *p* = 0.5325, *n* = 701 sites within 52 positions and 23 rats. AAF: interaction *F*_(4,253.2)_ = 1.74, *p* = 0.1414. Main effect of Group *F*_(1,34.73)_ = 3.00, *p* = 0.0920, *n* = 292 sites within 47 positions and 22 rats. VAF: interaction *F*_(4,317.9)_ = 0.13, *p* = 0.9698. Main effect of Group *F*_(1,37.34)_ = 0.09, *p* = 0.7683, *n* = 342 units within 45 positions and 23 rats). As a result, we performed two-way ANCOVAs with intensity and latency as covariates to determine whether the tone-evoked firing rate differed between experimental groups controlling for these two variables. We did not find a significant difference between groups for any CF bin in A1 or VAF (mixed effects two-way ANCOVAs with Group and Bin as factors. A1: interaction *F*_(4,3662)_ = 0.35, *p* = 0.8456. Main effect of Group *F*_(1,50.68)_ = 0.01, *p* = 0.9073, *n* = 3,697 observations within 52 positions and 23 rats. VAF: interaction *F*_(4,1937)_ = 1.38, *p* = 0.2393. Main effect of Group *F*_(1,40.82)_ = 0.10, *p* = 0.7516, *n* = 1,965 observations within 45 positions and 23 rats). In AAF, however, we observed a significantly higher tone-evoked firing rate for the 3.5 kHz, 7 kHz, and 38 kHz bins (mixed effects two-way ANCOVAs with Group and Bin as factors. Interaction *F*_(4,1398)_ = 9.02, *p* < 0.0001 followed by simple main effects for 3.5 kHz *F*_(1,60.06)_ = 4.39, p 0.0403, 7 kHz *F*_(1,67.83)_ = 4.99, *p* = 0.0288, and 38 kHz *F*_(1,47.66)_ = 8.03, *p* = 0.0067. No other CF bins were significant *F*_(1,51.18–60.75)_ ≤ 0.23, both *p* ≥ 0.63, *n* = 1,431 observations within 47 positions and 22 rats) For all of the ANCOVAs above, intensity (*F*_(1,1383–3646)_ ≥ 35.56, all *p* ≤ 0.0001) and latency (*F*_(1,1406–3666)_ ≥ 78.36, all *p* ≤ 0.0001) remained significant factors. These results show that after sound exposure, tone-evoked firing rate was greater within AAF for neurons tuned to a broad range of frequencies.

**Figure 2:**
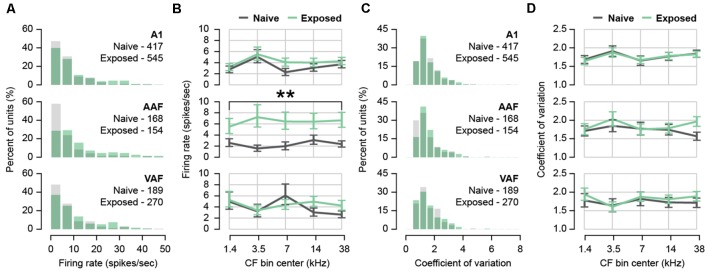
Effect of sound exposure on spontaneous firing rate and burst firing. **(A)** Histogram of firing rates for each auditory field. N units per field and group in inset. **(B)** Back-transformed mean firing rate with CF in five frequency bins for each auditory field. **(C)** Histogram of coefficient of variation (CV) for each auditory field. N units per field and group in inset. **(D)** Back-transformed mean CV in five frequency bins for each auditory field. **p < 0.01. Error bars represent SEM. A1, primary auditory field; AAF, anterior auditory field; VAF, ventral auditory field. See Table 1 for number of rats, recording positions, and units per auditory field and group.

Tinnitus and hyperacusis are associated with higher spontaneous firing rates (Wang et al., 2011; Kaltenbach, 2011), and tinnitus in particular is associated with more burst firing in the auditory pathway including the auditory thalamus (Kalappa et al., 2014) and auditory cortex (Syka and Rybalko, 2000; Noreña and Eggermont, 2003). From 5-min-long recordings of spontaneous activity during silence, we computed the spontaneous firing rate and inter-spike intervals (ISIs) of single-unit activity (Figure 2). Each sorted unit was assigned an auditory field and CF based on sound-evoked responses in the same recording position resulting in a total of 1,743 units from A1, AAF, and VAF combined. The number of units included in each auditory field and group is listed in Table 1. Because the distribution of firing rates was positively skewed (Figure 2A), we applied a natural logarithmic transform before statistical analyses. Back-transformed means are plotted in Figure 2B. The average spontaneous firing rates of our naïve animals were as follows: A1 = 5.33 ± 0.33, AAF = 4.77 ± 0.58, VAF = 5.67 ± 0.57 spikes/second. After sound exposure, we did not observe any difference in spontaneous firing rates in A1 or VAF regardless of CF bin (mixed effects two-way ANOVAs with Group and Bin as factors. A1: Interaction *F*_(4,932.2)_ = 0.85, *p* = 0.4951. Main effect of Group *F*_(1,40.85)_ = 1.04, *p* = 0.3134, *n* = 962 units within 44 positions and 23 rats. VAF: Interaction *F*_(4,442.9)_ = 1.65, *p* = 0.1596. Main effect of Group *F*_(1,37.86)_ = 0.34, *p* = 0.5626, *n* = 459 units within 37 positions and 22 rats). In AAF, on the other hand, sound exposure led to a significant and uniform increase in firing rate for all CF bins (mixed effects two-way ANOVA with Group and Bin as factors. Interaction *F*_(4,304.7)_ = 1.06, *p* = 0.3759. Main effect of Group *F*_(1,39.57)_ = 14.67, *p* = 0.0004, *n* = 322 units within 37 positions and 22 rats). The increased spontaneous firing rate in AAF indicates strong, tuning-independent hyperactivity resulting from sound exposure.

The ISI coefficient of variation (CV) was used to estimate the bursting activity of auditory neurons. This measure was obtained by dividing the standard deviation of each unit’s ISI distribution by its mean (Longenecker and Galazyuk, 2016). A high CV indicated more irregular spiking intervals, suggestive of bursting. Again, the distribution of CVs was positively skewed (Figure 2C) so a natural logarithmic transform was applied before statistical analyses and back-transformed means are plotted in Figure 2D. We did not observe any difference in the average CV of any field after sound exposure (mixed effects two-way ANOVAs with Group and Bin as factors. A1: Interaction *F*_(4,932.3)_ = 0.07, *p* = 0.9908. Main effect of Group *F*_(1,39.63)_ = 0.01, *p* = 0.9126, *n* = 962 units within 44 positions and 23 rats. AAF: Interaction *F*_(4,306.8)_ = 1.40, *p* = 0.2325. Main effect of Group *F*_(1,36.99)_ = 0.78, *p* = 0.3842, *n* = 322 units within 37 positions and 22 rats. VAF: Interaction *F*_(4,433.8)_ = 0.32, *p* = 0.8627. Main effect of Group *F*_(1,33)_ = 0.30, *p* = 0.5886, *n* = 459 units within 37 positions and 22 rats). From this, we concluded that burst firing was unchanged in the auditory cortex following sound exposure.

**Figure 3:**
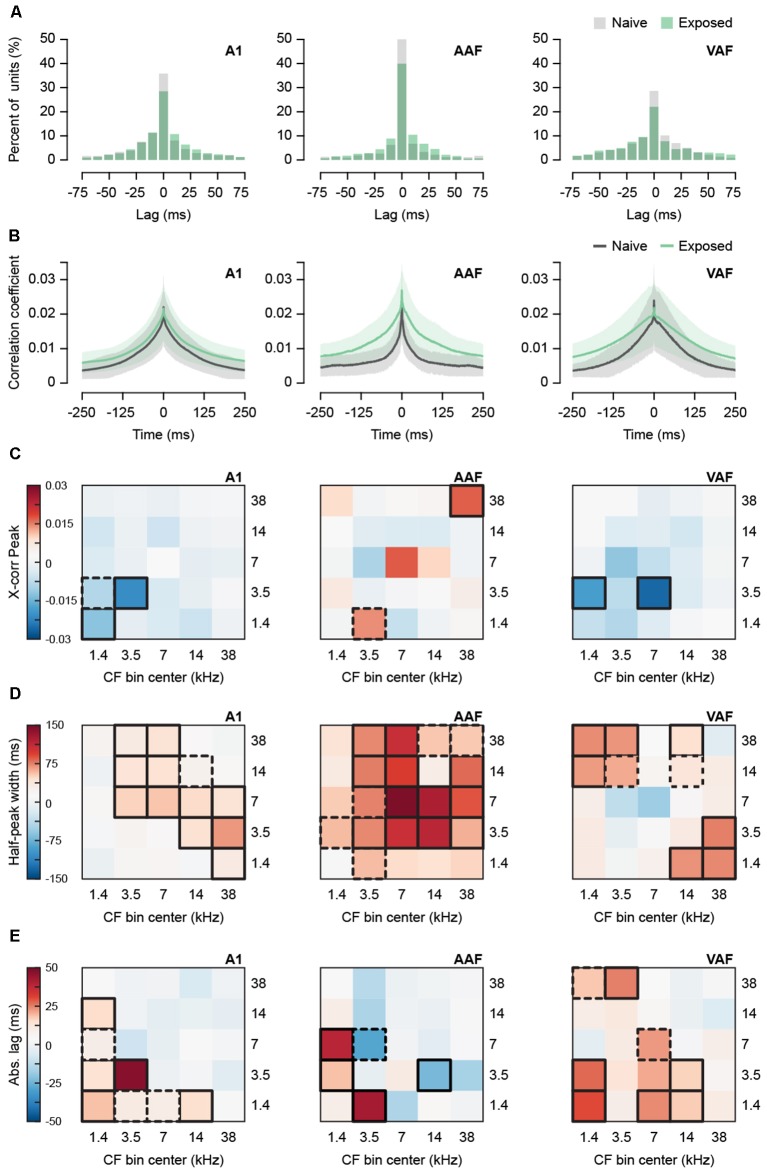
Effect of sound exposure on spontaneous synchronization. **(A)** Histogram of lag times of the peak of the cross-correlogram for all recorded unit pairs in A1, AAF, and VAF. Data for lag times outside −75 and 75 are not shown. **(B)** Average cross-correlogram for all unit pairs detected in separate channels in A1, AAF, and VAF. Shaded region represents SEM. **(C)** Subtracted (Exposed—Naïve) difference between average peak correlation coefficient for unit pairs with CF in five frequency bins. **(D)** Subtracted (Exposed—Naïve) difference between average half-peak width for unit pairs with CF in five frequency bins. **(E)** Subtracted (Exposed—Naïve) difference between average time lag in absolute values of the peak of the cross-correlogram. Bolded boxes are significant with *p* < 0.05. Dashed boxes represent *p*-values < 0.10. A1, primary auditory field; AAF, anterior auditory field; VAF, ventral auditory field; CF, characteristic frequency. N unit pairs per auditory field and group: Naïve A1 3,614; AAF 746; VAF 1,092. Exposed A1 6,116; AAF 733; VAF 1,707. See Table 1 for number of rats, recording positions, and units per auditory field and group.

Tinnitus has also been associated with hypersynchronization in animal models. Hypersynchronization typically appears immediately after noise trauma in a frequency-specific manner (Eggermont and Roberts, 2004) and is evidence of increased connectivity, either thalamocortical or corticocortical, between neurons. To assess whether the Exposed group displayed hypersynchronization, we calculated normalized cross-correlograms between single-unit pairs recorded in silence (Figure 3). From the 1,743 units detected above, we identified 16,441 unit pairs in separate channels. We limited our analysis to pairs with a peak between −150 and 150 ms, falling within approximately ±2.3 standard deviations of the mean peak, resulting in a total of 14,008 unit pairs for all fields. Figure 3A shows histograms of the cross-correlogram peak lag times demonstrating that peaks tend to fall near 0 ms and Figure 3B shows the average cross-correlogram for all pairs in each field. The peak value of the cross-correlogram tended to decrease with greater inter-unit distance, *r* = −0.24, *p* < 0.0001, *n* = 14,008 pairs, as well as greater ΔCF, *r* = −0.24, *p* < 0.0001, *n* = 14,008 pairs. Distance and ΔCF were positively related, *r* = 0.42, *p* < 0.0001, *n* = 14,008 pairs. As a result, we performed mixed-effects two-way ANCOVAs with distance as a covariate to determine whether the peak correlation coefficient differed between exposure groups while controlling for differences in inter-unit distance (Figure 3C). As the distribution of peaks was positively skewed, we multiplied the data, originally on a 0–1 scale, by 100 and applied a natural logarithmic transform before statistical analyses. In Figure 3C, the difference between back-transformed group means for each CF bin combination is depicted by a heatmap. The interaction was significant for A1, AAF, and VAF (mixed effects two-way ANCOVAs with Group and Combined CF Bin as factors and Distance as covariate: A1 *F*_(24,9632)_ = 5.35, *p* < 0.0001, AAF *F*_(24,1407)_ = 2.16, *p* = 0.0009, VAF *F*_(24,2730)_ = 1.88, *p* = 0.0060). The simple main effect of Group was evaluated over each level of Combined CF Bin and the significant comparisons are outlined in bold on the heatmap in Figure 3C. Distance remained a significant covariate in each ANCOVA (A1 *F*_(1,9650)_ = 358.29, *p* < 0.0001, AAF *F*_(1,1406)_ = 47.43, *p* < 0.0001, VAF *F*_(1,2740)_ = 47.48, *p* < 0.0001). From the heatmaps, we observed few significant differences in synchronization strength. Sound-exposed A1 and VAF tended to have shorter cross-correlograms for most frequency combinations, with peak values being significantly smaller for low-to-medium frequency combinations only. In AAF, differences with respect to naïve animals were less consistent. Only synchronization between unit pairs where both units had CFs in the 38 kHz bin was significantly greater.

The strength of synchronization can also be estimated by the width of the cross-correlogram, with wider functions representing greater synchronization at longer lag times. The width at half-height of each peak was compared between exposure groups as a function of CF bin (Figure 3D). Width could not be computed for 38 pairs for which the function did not dip below half-height, resulting in 13,970 analyzed pairs. Width was found to weakly but significantly increase with inter-unit distance, *r* = 0.03, *p* = 0.0009, *n* = 13,970 pairs, and ΔCF, *r* = 0.03, *p* = 0.0013, *n* = 13,970. However, distance did not remain significant when included as a covariate for any field (mixed effects two-way ANCOVAs with Group and Combined CF Bin as factors and Distance as covariate. Effect of Distance: A1 *F*_(1,9651)_ = 0.22, *p* = 0.6381, AAF *F*_(1,1403)_ = 0.003, *p* = 0.9545, VAF *F*_(1,2739)_ = 0.41, *p* = 0.5239). As a result, we removed the covariate and performed mixed-effects two-way ANOVAs. The interaction was significant for A1, AAF, and VAF (mixed effects two-way ANOVA with Group and Combined CF Bin as factors: A1 *F*_(24,9499)_ = 4.12, *p* < 0.0001, AAF *F*_(24,1422)_ = 2.60, *p* < 0.0001, VAF *F*_(24,2741)_ = 1.67, *p* = 0.0214). The simple main effect of Group was evaluated over each level of Combined CF Bin and the significant comparisons are outlined in bold on the heatmap in Figure 3D. In the heatmaps, we observed clear wider cross-correlograms in the sound-exposed A1, AAF, and VAF. In A1, this trend showed units in low-to-mid frequency bins having wider cross-correlograms with units in the highest frequency bins. In AAF, almost every frequency bin combination tended to have wider cross-correlograms, with significant differences in the mid-to-high frequency combinations, and notably with the 7 kHz bin showing the greatest increase in width. Interestingly, VAF showed an opposite trend, where only the lowest frequency bins had significantly wider cross-correlograms when paired with the highest frequency bins. The mid-range bins, including 7 kHz, showed either no change in width or a slight decrease in width for VAF.

A greater average cross-correlogram width could result from either more pairs with broad cross-correlograms or more pairs with off-centered peaks. To investigate the contribution of pairs with off-centered peaks to the wider cross-correlograms we observed in each field, we conducted mixed-effects two-way ANCOVAs with distance as a covariate on the absolute lag of the peak of the cross-correlogram (Figure 3E). Distance was positively correlated with absolute lag, *r* = 0.23, *p* < 0.0001 *n* = 14,008 pairs. The interaction between exposure group and CF bin was significant for A1, AAF, and VAF (mixed effects two-way ANCOVA with Group and Combined CF Bin as factors and Distance as covariate: A1 *F*_(24,9499)_ = 4.12, *p* < 0.0001, AAF *F*_(24,1422)_ = 2.60, *p* < 0.0001, VAF *F*_(24,2741)_ = 1.67, *p* = 0.0214). The simple main effect of Group was evaluated over each level of Combined CF Bin and the significant comparisons are outlined in bold on the heatmap in Figure 3E. Distance remained a significant covariate for all three fields (A1 *F*_(1,9679)_ = 305.61, *p* < 0.0001, AAF *F*_(1,1427)_ = 7.50, *p* = 0.0062, VAF *F*_(1,2748)_ = 45.29, *p* < 0.0001). The heatmaps revealed mostly increases in absolute lag for the Exposed group, suggestive of a greater number of off-centered peaks. However, in A1 and AAF, the CF bins with greater absolute lag did not correspond with those that showed the broadest widths in Figure 3D. This suggests that a greater number of broadly synchronized unit pairs contribute to the wider cross-correlograms in these fields. In VAF, some CF bins with wider cross-correlograms corresponded with bins that also had greater absolute lag, indicating a mixed contribution between broader synchronization and off-centered peaks.

### Behavioral Correlates of Sound Exposure

A common behavioral measure for detecting tinnitus in rodents is gap-prepulse inhibition of the acoustic startle response (GPIAS), in which a short silent gap within a background sound carrier reduces the magnitude of a rodent’s involuntary startle to a subsequent loud noise burst (Brozoski and Bauer, 2016). Impaired GPIAS is considered evidence of tinnitus in rodents, meaning that the gap is less effective at reducing the startle response, possibly because the presence of tinnitus interferes with the ability to hear silence. This test is usually accompanied by a similar measure called prepulse inhibition (PPI) of the acoustic startle response. PPI has been proven useful in characterizing hyperacusis and hypoacusis, since a short tonal stimulus will either enhance or dampen inhibition of the startle response in rodents with hyper- or hypoacusis, respectively (Carlson and Willott, 1996; Turner and Parrish, 2008; Turner and Larsen, 2016; Pienkowski, 2018). To investigate whether sound exposure could have altered these behavioral measures, we performed GPIAS and PPI testing on two additional groups of naïve (Naïve-BEH, *N* = 12) and exposed (Exposed-BEH, *N* = 12) rats.

**Figure 4:**
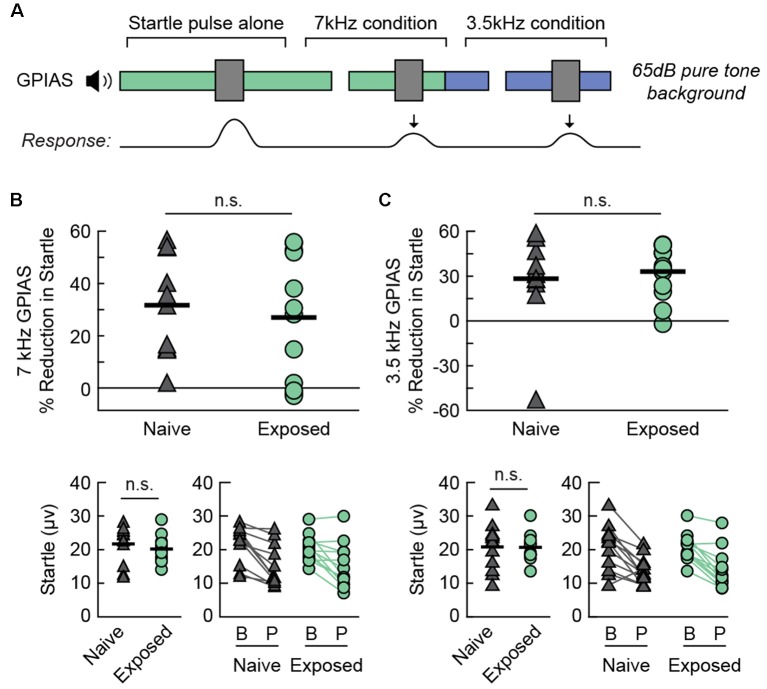
Sound exposed rats demonstrate no change in GPIAS. **(A)** A schematic drawing of the behavioral protocol. Testing takes place in the presence of a 65 dB SPL continuous pure tone (3.5 or 7 kHz). A 30 ms silent gap prepulse preceding the startle sound (40 ms white noise burst, 120 dB SPL) reduces the magnitude of the acoustic startle response. **(B)** Percent GPIAS (Top), baseline startle response (Bottom Left), and comparison of startle response between Baseline (B) and Prepulse (P) trials (Bottom Right) in the presence of a 7 kHz pure tone background. Lines connect responses from the same animal. **(C)** Percent GPIAS (Top), baseline startle response (Bottom Left), and comparison of startle response between Baseline (B) and Prepulse (P) trials (Bottom Right) in the presence of a 3.5 kHz pure tone background. Lines connect responses from the same animal. Ns, not significant. *N* rats per group: 12 Naive, 12 Exposed. GPIAS, Gap-Prepulse Inhibition of the Acoustic Startle response.

We found that the Exposed-BEH group did not differ from Naïve-BEH in GPIAS (Figure 4). A schematic of the behavioral protocol for GPIAS is presented in Figure 4A. We hypothesized that a deficit in inhibition of the acoustic startle response would be specific to the 7 kHz exposure frequency. To test this, we performed testing in the presence of either a 7 kHz pure tone background or a 3.5 kHz pure tone background with the order of testing counterbalanced between pairs. First, we confirmed that the magnitude of the response to the startle pulse alone was not significantly different between groups for either pure tone condition (7 kHz: two-tailed paired *t*-test *t*_(11)_ = −0.81, *p* = 0.4372, Cohen’s dav −0.32; 3.5 kHz: two-tailed paired *t*-test *t*_(11)_ = −0.08, *p* = 0.9371, Cohen’s dav −0.03, *n* = 12 pairs, Figures 4B,C, bottom left). Next, we computed the percent reduction in the startle response when the startle pulse was preceded by a silent gap. We found that the average reduction in startle did not differ between groups for either the 7 kHz (one-tailed paired *t*-test *t*_(11)_ = −0.45 *p* = 0.3303, Cohen’s dav −0.22, *n* = 12 pairs, Figure 4B) or the 3.5 kHz (one-tailed paired *t*-test *t*_(11)_ = 0.44 *p* = 0.6656, Cohen’s dav 0.20, *n* = 12 pairs, Figure 4C) condition. From these results, we concluded that sound exposure did not lead to behavioral evidence of tinnitus in any frequency tested.

**Figure 5:**
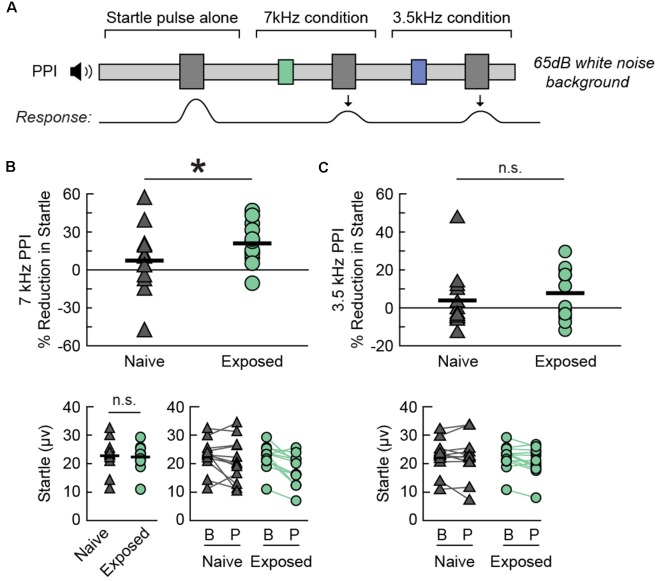
Sound exposed rats demonstrate a frequency-specific enhancement in PPI. **(A)** A schematic drawing of the behavioral protocol. Testing takes place in the presence of a continuous 65 dB SPL background noise. A 20 ms tone pip (3.5 or 7 kHz, 75 dB SPL) preceding the startle sound (40 ms white noise burst, 120 dB SPL) reduces the magnitude of the acoustic startle response. **(B)** Percent PPI (Top), baseline startle response (Bottom Left), and comparison of startle response between Baseline (B) and Prepulse (P) trials (Bottom Right) when the prepulse is a 7 kHz tone. Lines connect responses from the same animal. **(C)** Percent PPI (Top) and comparison of startle response between Baseline (B) and Prepulse (P) trials (Bottom) when the prepulse is a 3.5 kHz tone. Lines connect responses from the same animal. Note that the same baseline startle values were used for computing PPI in **(B,C)**. *p < 0.05, ns, not significant. *N* rats per group: 12 Naive, 12 Exposed. PPI, Prepulse Inhibition of the acoustic startle response.

We observed an enhancement in PPI for the Exposed-BEH group when the prepulse was a 7 kHz tone (Figure 5). A schematic of the behavioral protocol for PPI is presented in Figure 5A. Of note, a magnified response to the startle pulse alone is also sometimes taken as evidence of hyperacusis (Chen et al., 2013), but we hypothesized that an improvement in inhibition of the acoustic startle response would be specific to the 7 kHz exposure frequency. As a result, we performed PPI testing using either a 7 kHz or 3.5 kHz pure tone prepulse with the order of 7 kHz prepulse, 3.5 kHz prepulse, and no prepulse trials randomly interleaved within a single testing session. Testing took place in the presence of a 65 dB white noise background. We observed that the magnitude of the response to the startle pulse alone was not significantly different between groups (paired *t*-test *t*_(11)_ = −0.21, *p* = 0.8343, Cohen’s dav −0.09, *n* = 12 pairs, Figure 5B, bottom left). Next, we compared the average percent reduction in the startle response when the startle pulse was preceded by a prepulse tone. We found that the average reduction in startle was significantly greater for the Exposed-BEH group when the prepulse was a 7 kHz tone (one-tailed paired *t*-test *t*_(11)_ = 2.69 *p* = 0.0105, Cohen’s dav 0.63, *n* = 12 pairs, Figure 5B) but not when the prepulse was a 3.5 kHz tone (one-tailed paired *t*-test *t*_(11)_ = 0.66 *p* = 0.2621, Cohen’s dav 0.29, *n* = 12 pairs, Figure 5C) condition. Our positive findings remained significant when adjusting the alpha value to account for three comparisons using either the Bonferroni or Holms-Bonferroni correction (both *α* = 0.0167). From these results, we concluded that Exposed-BEH exhibited behavioral evidence of hyperacusis for the 7 kHz frequency.

## Discussion

Passive exposure to moderate-intensity broadband white noise can be used to open a CP window for frequency tuning in the adult rat auditory cortex, allowing for subsequent frequency-specific reorganization of the tonotopic map. This phenomenon could have profound implications for plasticity-based neurotherapeutics that aim to improve learning and memory or treat disorders of plasticity through non-invasive means. However, frequency-specific tonotopic map expansions and regional changes in excitability have also been described as symptoms of tinnitus and hyperacusis in animal models, leading us to wonder whether noise exposure could increase the risk of developing one or both of these disorders. In the present investigation, we extended previous studies by examining the effects of noise and tone pip exposure on secondary auditory fields and carried out novel experiments to determine whether sound-exposed animals display evidence of tinnitus or hyperacusis.

As in previous studies (Zhou et al., 2011; Thomas et al., 2018), we observed map expansion in the A1 of adult rats passively exposed to moderate-intensity broadband white noise followed by tone pips with no elevation in cortical thresholds. We also showed for the first time that a CP-like window is also opened in VAF as demonstrated by map expansion in this field accompanied by broader receptive field bandwidths for 7 kHz-tuned neurons. Apart from map expansion, however, we observed few changes in spontaneous activity or auditory processing in the A1 and VAF of exposed animals. In contrast, we observed strong evidence of hyperactivity in AAF, where there was no map expansion. This included an overall increased spontaneous firing rate, stronger tone-evoked firing rates and narrower receptive field bandwidths for a range of frequencies, and a lower cortical threshold for 7 kHz-tuned neurons. Despite changes in AAF affecting multiple iso-frequency bands, the band corresponding to 7 kHz showed changes consistent with heightened sensitivity in all of our measures. Our behavioral results also pointed to enhanced sensorimotor gating for the 7 kHz frequency, since exposed rats had improved PPI when the prepulse was a 7 kHz pure tone. Taken together, our findings point to hyperacusis for the 7 kHz frequency in sound-exposed animals.

We expected hypersynchronization to accompany map expansion given the close link between receptive field overlap and neural synchronization (Noreña and Eggermont, 2006; Eggermont, 2007; Kilgard et al., 2007). However, we did not observe clear hypersynchronization in any field. The absence of this relationship could be due to the unique manner in which noise induces plasticity. Noise exposure on its own produces lasting desynchronization with shorter cross-correlogram peaks in A1 (Zhou et al., 2011; Kamal et al., 2013; Thomas et al., 2018). The prevalence of lower peaks and broader cross-correlogram widths observed in A1 and VAF could be consistent with these earlier findings, assuming partial recovery of desynchronization potentially hastened by tone pip exposure. The broader widths that we observed in AAF centered on 7 kHz are likely the combined result of greater disinhibition and increased firing, since secondary effects independent of connectivity can also affect the width of the cross-correlogram. These include firing patterns intrinsic to each neuron, such as burst firing, and global oscillations (Eggermont and Smith, 1996; Nowak and Bullier, 2005). Although cross-correlograms were normalized with respect to firing rate and significant differences in burst firing measured by CV were not observed, a higher firing rate could increase the impact of secondary effects on cross-correlogram width. Clear evidence of disinhibition or changes in firing were not observed in A1 or VAF in the present study, therefore assumptions about the origin of broader cross-correlogram widths beyond residual effects of noise exposure remain speculative.

The role of the auditory cortex in generating tinnitus and hyperacusis has not been fully established. Although changes in neural activity related to hearing loss begin in the auditory nerve and cochlear nucleus, individuals with clinically normal audiograms can also report these percepts, and electrophysiological signatures of each condition have been reported in cortex in animal models. In addition, studies have primarily identified A1 and the auditory thalamus (medial geniculate body, MGB) as sites of experience-dependent plasticity following non-traumatic passive sound exposures (Pienkowski and Eggermont, 2011; Lau et al., 2015; Pienkowski, 2018), revealing a possible mechanism by which passive experience could lead to changes in auditory processing in the absence of hearing loss. Here, we observed a significant difference between the electrophysiological response properties of A1 and AAF following noise exposure that may suggest a causal role for AAF in the generation of hyperacusis. Whereas A1 has been studied extensively in the context of passive sound exposure, much less is known about how AAF adapts to such experiences. Sparse findings demonstrate asymmetric plasticity in each field despite both receiving direct inputs from the ventral MGB and displaying similar tone-evoked response properties (Polley et al., 2007). Takahashi et al. (2006) found that A1 responses of juvenile mice were more potentiated than those of AAF following exposure to an amplitude-modulated tone for 4–5 weeks but did not observe an over-representation of the exposure frequency in either field. A recent study documented differences in parvalbumin positive (PV+) interneuron and peri-neuronal net (PNN) densities in A1 and AAF following 70 dB SPL broadband noise exposure in mice during the first month of life (Reinhard et al., 2019). Noise exposure decreased the density of PNNs in A1 but not AAF, showing that inhibitory elements can be differently regulated across these two fields. Given the preliminary nature of our study, further studies should be undertaken to understand the potential mechanisms by which AAF could contribute to hyperacusis.

The sound exposure paradigm used to induce 7 kHz map expansion consisted of two distinct components: white noise exposure and 7 kHz tone pip exposure. On its own, chronic exposure to moderate-intensity white noise has been shown to lead to tonotopic disorganization, broadened receptive field bandwidths, decreased neural synchronization, and disrupted temporal processing. These plastic changes develop whether the noise is present for 2 weeks (Thomas et al., 2018), 6–8 weeks (Zhou et al., 2011; Kamal et al., 2013), or on a 10-h-per-day schedule (Zhou and Merzenich, 2012). As long as the noise is broadband, its effects are non-frequency-specific as illustrated by comparison with band-limited noise exposure (de Villers-Sidani et al., 2008). In the present study, the most prominent electrophysiological measure that was affected in a non-frequency-specific manner was the increased spontaneous firing rate in AAF, and it is possible that this change was driven primarily by white noise exposure. Exposure to non-traumatic white noise has been scarcely studied in the context of PPI or GPIAS, especially in contrast to traumatic noise exposures. One exception is a recent study that found that band-limited noise exposure did not produce either hyper- or hypoacusis in mice exposed for 3 months (Pienkowski, 2018).

Taken alone, exposure to pure tones has not been shown to induce strong cortical plasticity leading to map expansion or altered discrimination abilities for the exposure frequency in adult rodents (Zhou et al., 2011; Blundon et al., 2017). This is consistent with the view that the mature cortex is largely resistant to change based on passively experienced stimuli (Keuroghlian and Knudsen, 2007). However, extensive research performed in cat auditory cortex has shown convincingly that band-limited tone pip ensembles can lead to frequency-specific changes in auditory responsiveness after chronic exposure (Pienkowski and Eggermont, 2009, 2010; Pienkowski et al., 2011). Specifically, cortical regions tuned to the exposure frequency range show reduced responsiveness while cortical regions outside the exposure frequency range show increased responsiveness. This suggests that there could be detectable differences in the electrophysiological properties of the auditory cortex after tone pip exposure that could also alter behavioral responses to PPI or GPIAS. However, extrapolating from these results one would expect animals exposed to 7 kHz to display hypoacusis for this frequency. Consequently, we do not believe that tone pip exposure on its own would cause increased cortical sensitivity to 7 kHz.

A limitation of the present study is that in the interest of reducing the number of animals used, the electrophysiological data for the Exposed group came from combining two groups of sound-exposed animals that underwent slightly different 7 kHz exposures (i.e., 7 kHz tone pips vs. 7–8.3 kHz tone pip clouds, see “Materials and Methods” section). It is likely that these exposures would produce different electrophysiological signatures. For example, we would expect tone pip clouds to lead to map expansion for a broader frequency range. To account for this, we used relatively coarse (≥1 octave) CF bins in our analysis so that the 7 kHz bin spanned 5–10 kHz and presumably encompassed all neurons that would have shifted their CFs to the exposure frequencies. However, the combined group contained a greater number of rats exposed to tone pip clouds (*n* = 9) than tone pips (*n* = 4), so it is possible that the average data is a better representation of the tone pip cloud exposure. The Exposed-BEH group, on the other hand, was not heterogeneous; every rat was exposed to the tone pip stimulus. As a result, the Exposed group used for electrophysiology is not a perfect analogue for the Exposed-BEH group. Furthermore, because the animals used for behavioral testing in our study were not the same animals that were used for electrophysiological recording, we were unable to correlate auditory response properties with PPI or GPIAS. This additionally prevents us from making any direct conclusions about cortical properties, such as degree of map expansion, that may have influenced inhibition of the acoustic startle response.

A second limitation is that classifying auditory fields based purely on functional characteristics will always result in an imperfect classification. It is possible that some cortical sites, especially those on borders with CF gradient reversals (such as A1 and AAF), were misclassified as being in neighboring fields. Of note, we were unable to distinguish VAF and SRAF based purely on functional properties and therefore pooled the data from these fields. These challenges are not unique to our study, and there is precedence for pooling VAF and SRAF with sparse datasets (Takahashi et al., 2011). Without accompanying anatomical tracer data or similar, conclusions about the response properties of any auditory field should only be drawn from multiple independent replications. Importantly, an experimenter blind to the identity of the experimental groups performed field classification for the present study. The average response properties reported for each field in Supplementary Figure S1 are in strong agreement with the published literature on the adult rat auditory cortex (Polley et al., 2007; Profant et al., 2013).

Finally, stress is another factor that could have played a role in our results, as it is known to affect PPI (Guercio et al., 2014). Importantly, chronic noise exposure, even at moderate intensities, is a known stressor for humans and animals and has a complex interplay with tinnitus, mostly exacerbating its symptoms (Eggermont, 2017a). The chronic sound exposures used in our study could have caused stress that could affect the acoustic startle response or PPI. The main argument against this, however, is that we did not observe differences in baseline startle response between exposed and unexposed animals. Furthermore, any stress-induced differences in PPI or GPIAS would likely not have been specific to the 7 kHz frequency.

In summary, our study examines the phenomenon of noise-induced map expansion and demonstrates that prolonged exposure to moderate-intensity noise could be considered a risk factor for hyperacusis in adulthood. Our results could have implications for noise levels presently deemed “safe” in occupational, private, and public settings (Pienkowski and Eggermont, 2012; Gourévitch et al., 2014; Eggermont, 2017b). Rather than suggesting that noise exposure should not be used for neurotherapeutic purposes, however, we would urge continued investigation into this subject. For one, sensorimotor gating measured by PPI is impaired in some neuropsychiatric disorders, most notably schizophrenia (Swerdlow et al., 2000; Swerdlow and Light, 2018). Noise-induced map expansion could thus be a way to target and reverse this specific preattentional deficit (Braff and Light, 2004). Additional candidate strategies to drive plasticity in a sensory-specific manner are vagus nerve stimulation paired with the presentation of pure tones (Engineer et al., 2011) and cognitive training programs designed to improve basic sensory processing (Cramer et al., 2011; Merzenich et al., 2014). Exciting or inhibiting specific brain areas through sensory experience is a more targeted and non-invasive means of driving plasticity than purely pharmaceutical strategies such as those presently used in the treatment of schizophrenia (Guercio et al., 2019) and dementia (Farlow and Cummings, 2007; Massoud and Léger, 2011). By focusing on “retuning” cortical maps, sensory deprivation or stimulation paradigms in other systems could potentially be developed to treat or reverse symptoms of sensory disorders such as phantom sensations or chronic pain (Flor et al., 2001; Tabot et al., 2015). Through both electrophysiological and behavioral measures, the results of our study suggest that map expansion induced by passive sound exposure opens windows of plasticity that can also be understood as windows of vulnerability. However, as our understanding of the rules that regulate plasticity and the opening and closure of CPs progress, our hope is that we will 1 day be able to harness them to treat a variety of brain disorders.

## Data Availability Statement

The datasets generated for this study can be obtained upon request from the study authors.

## Ethics Statement

The animal study was reviewed and approved by Montreal Neurological Institute Animal Care Committee.

## Author Contributions

GG and EV-S designed the study. GG, KD and MT performed the experiments. GG and KD obtained the behavioral data. MT obtained the electrophysiological data, analyzed the data, and wrote the first draft of the manuscript. All authors contributed to manuscript revision, read, and approved the submitted version.

## Conflict of Interest

The authors declare that the research was conducted in the absence of any commercial or financial relationships that could be construed as a potential conflict of interest.
