# Supplementary material for: Evidence of Hyperacusis in Adult Rats Following Non-traumatic Sound Exposure
Source: Front Syst Neurosci. 2019 Oct 23;13:55. doi: 10.3389/fnsys.2019.00055 (PMC6819503; doi:10.3389/fnsys.2019.00055)
Supplement: Supplementary file 1 [file Data_Sheet_1.pdf]

Supplementary Material

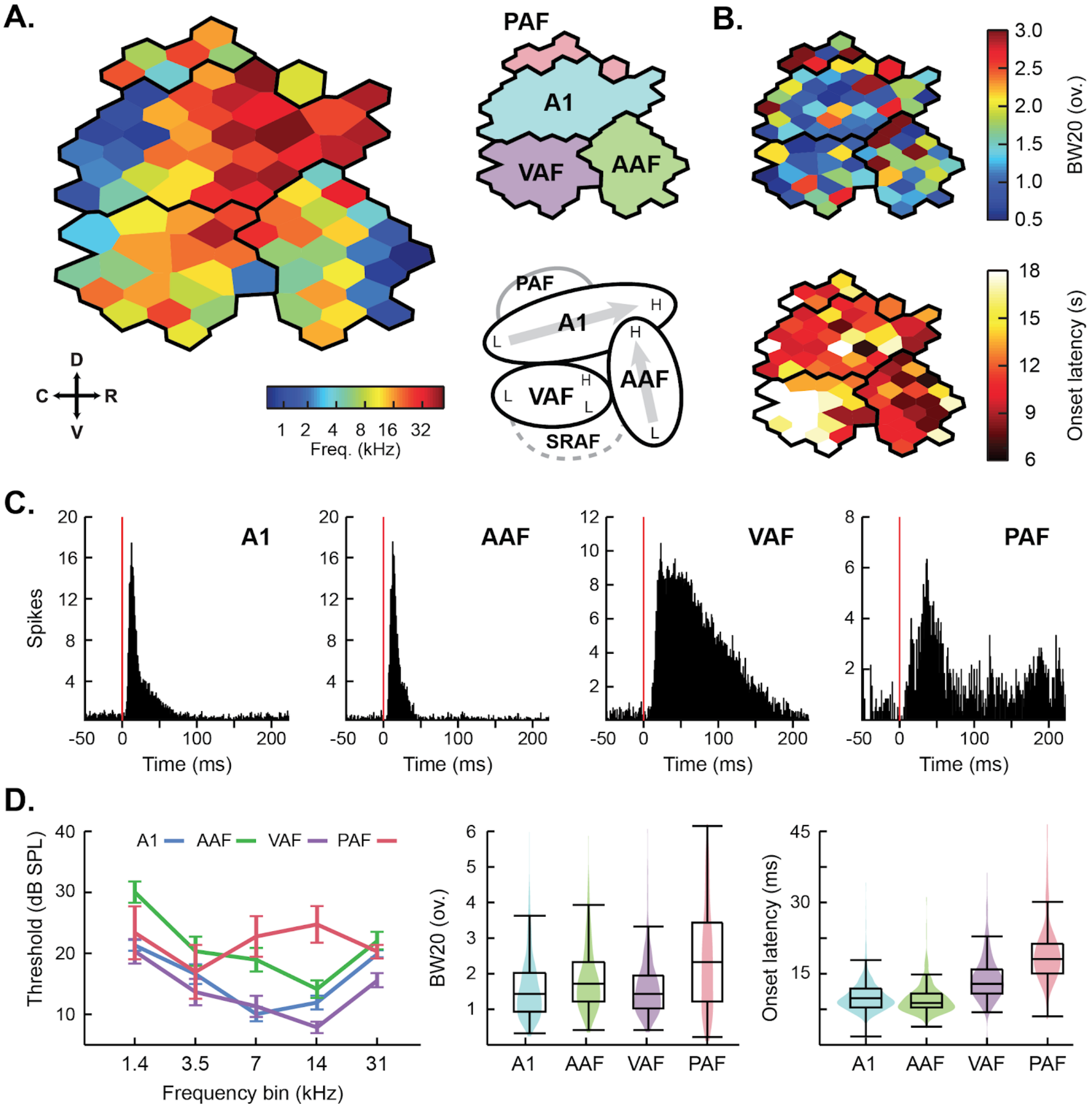

Supplementary Figure S1: *Classification of multiple fields within the rat auditory cortex.* **(A)** Example of auditory field classification for a tonotopic map from a representative rat with separate fields outlined in black (L = Tuning to low sounds, H = Tuning to high sounds). SRAF was not distinguishable from VAF in our data, so the presumed location of SRAF is represented by a dotted line. (**B)** Receptive field bandwidths 20dB SPL above threshold (top) and onset latencies (bottom) for the same rat as in A. (**C)** Average tone-evoked peri-stimulus time histograms (PSTH) for each field from a single rat. N sites per field: 26 A1, 7 AAF, 11 VAF, 2 PAF. **(D)** Average receptive field threshold (left), distribution of receptive field bandwidth 20dB SPL above threshold (middle), and distribution of onset latency (right) for 24 rats. N sites per field: 701 A1, 292 AAF, 342 VAF, 174 PAF. A1 = primary auditory cortex, AAF = anterior auditory field, VAF = ventral auditory field, PAF = posterior auditory field, SRAF = suprarhinal auditory field.

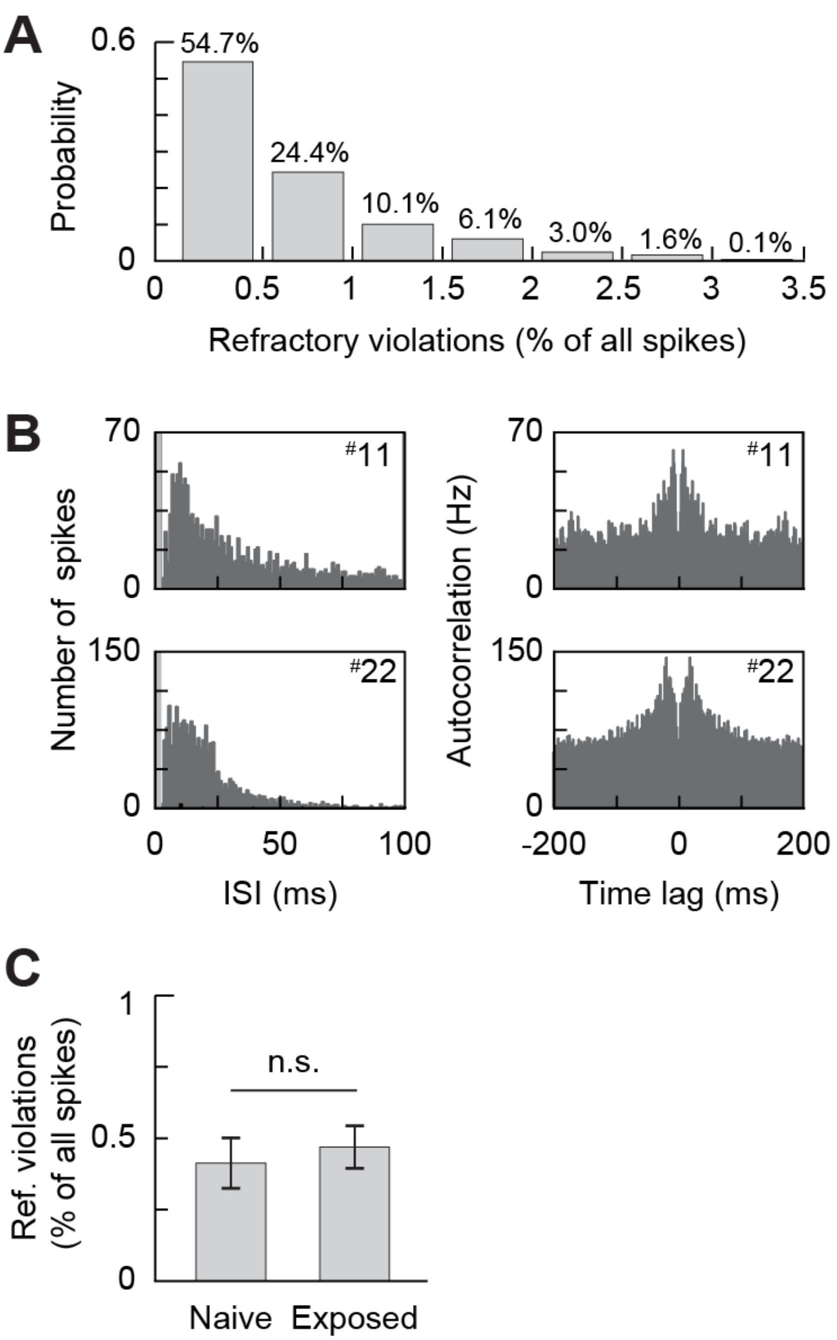

Supplementary Figure S2: *Quality assessments for spike sorting.* **(A)** Histogram of the fraction of refractory period violations out of all spikes for 2017 identified clusters in A1, AAF, VAF, and PAF. **(B)** Representative histograms for two isolated units (#11 and #22) of inter-spike interval (ISI) times (left) and autocorrelation functions (right). Bin widths are 1ms and gray shaded line represents the 2ms refractory period bin. **(C)** Mean fraction of refractory period violations out of all spikes for experimental groups. No difference was found between groups using a mixed-effects test $F(1,41.13) = 0.63$, $p = .4297$, $n = 2017$ units within 45 positions and 24 rats. Error bars represent S.E.M. Number of animals, recording positions, and units per group: Naïve 11,20,884, Exposed 13,25,1133.
